# Disordered semantic representation in schizophrenic temporal cortex revealed by neuromagnetic response patterns

**DOI:** 10.1186/1471-244X-6-23

**Published:** 2006-05-23

**Authors:** Andreas Löw, Brigitte Rockstroh, Thomas Elbert, Yaron Silberman, Shlomo Bentin

**Affiliations:** 1 Department of Psychology, University of Konstanz, 78457 Konstanz, Germany; 2 Department of Psychology, Hebrew University of Jerusalem, Jerusalem, 91905, Israel

## Abstract

**Background:**

Loosening of associations and thought disruption are key features of schizophrenic psychopathology. Alterations in neural networks underlying this basic abnormality have not yet been sufficiently identified. Previously, we demonstrated that spatio-temporal clustering of magnetic brain responses to pictorial stimuli map categorical representations in temporal cortex. This result has opened the possibility to quantify associative strength within and across semantic categories in schizophrenic patients. We hypothesized that in contrast to controls, schizophrenic patients exhibit disordered representations of semantic categories.

**Methods:**

The spatio-temporal clusters of brain magnetic activities elicited by object pictures related to super-ordinate (flowers, animals, furniture, clothes) and base-level (e.g. tulip, rose, orchid, sunflower) categories were analysed in the source space for the time epochs 170–210 and 210–450 ms following stimulus onset and were compared between 10 schizophrenic patients and 10 control subjects.

**Results:**

Spatio-temporal correlations of responses elicited by base-level concepts and the difference of within vs. across super-ordinate categories were distinctly lower in patients than in controls. Additionally, in contrast to the well-defined categorical representation in control subjects, unsupervised clustering indicated poorly defined representation of semantic categories in patients. Within the patient group, distinctiveness of categorical representation in the temporal cortex was positively related to negative symptoms and tended to be inversely related to positive symptoms.

**Conclusion:**

Schizophrenic patients show a less organized representation of semantic categories in clusters of magnetic brain responses than healthy adults. This atypical neural network architecture may be a correlate of loosening of associations, promoting positive symptoms.

## Background

The loosening of associations, defined by Bleuler [1] as abnormal linking of thoughts and dissociation between thoughts, emotions and behavior, has been considered a key feature of schizophrenic psychopathology, and has been assumed to mediate symptoms like thought disorders, hallucinations and delusions, and even inadequate affect. Commonly, thought disorders have been related to structural [2] and functional [3] abnormalities in the left temporal lobe. Although currently debated [4], previous studies reported higher than normal priming effects (e.g. for remote associated words [5] in schizophrenic patients). Results from neuropsychological studies indicate that schizophrenia patients show differences from controls in elaborative, organizational memory processes [6,7].

These findings suggest an insufficiently differentiated associative (semantic) network and further-reaching spreading activation within and among cortical neuronal networks [5]. Since dopamine [8] and acetylcholine [9] may influence cortical-map plasticity, an imbalance between dopaminergic and cholinergic systems, as assumed in schizophrenia [10], was discussed to account for the arbitrary spreading of activation and the formation of disordered associative networks.

Models of semantic processing assume networks of interconnected representations of object features. Following Hebb's model, neural networks are formed by synchronous neural activity during behaviorally relevant tasks. Therefore, Hebbian learning may account for the assignment of objects to distinct categories according to their physical properties and probabilities of co-occurrence.

Neuroimaging studies have delineated brain structures that are involved in the recognition and categorization of objects in primates as well as in humans [11,12]. These might be the neural basis for the formation of the semantic networks. Hence the identification of semantic categorization in the brain provides an approach to assess the formation and strength of associative networks in schizophrenic patients and in healthy individuals. Indeed, structural and functional abnormalities in brain areas involved in object representation have been reported in schizophrenia [2,13-15]. Thus, deficient object categorization might be a consequence of deficient association formation in schizophrenia.

In an earlier study, we examined semantic categorization in normal subjects, considering categorization as prerequisite for a meaningful organization of semantic knowledge [16]. Clustering magnetic brain responses to various stimuli disclosed association among base-level but distinction among super-ordinate object categories. For instance, the spatio-temporal correlation of brain activity elicited by base-level concepts was greater within than across super-ordinate categories in the right temporal lobe around 200 ms post-stimulus onset, and in the left temporal lobe around 400 ms [16]. In the present study, we compared categorical clustering results obtained from subjects in this previous study with a sample of patients with schizophrenia to uncover potential differences or weaknesses of semantic associative network in these patients. We hypothesized that categorical clusters would be less distinct in the brain responses of schizophrenic patients and, given that semantic categories are functionally organized mainly in the temporal lobes, the differences should be most prominent in activities generated in these brain regions.

## Methods

### Ethical approval

The study protocol was approved by the Ethical Review Board of the University of Konstanz and written informed consent was obtained from all participants after being provided with complete information about procedure and measurements.

### Subjects

Data of ten in-patients with DSM-IV diagnoses of schizophrenia (3 females, mean age: 26.4 +/- 5.8 years, paranoid-hallucinatory or disorganized subtype) were compared to the data of ten healthy subjects (5 females, mean age: 26.1 +/- 3.2 years; AGE: GROUP: t(18) = 0.14, n.s.) described in our previous study [16]. Patients were admitted to the university ward at the local Center of Psychiatry Reichenau. Diagnoses were given by the psychiatrists or psychologists in charge on the basis of an interview, DSM-IV criteria and the Present State Examination (PSE). The psychopathological status of each patient was assessed on the day of the neurophysiological investigation by the psychologist or psychiatrist in charge by means of the Positive and Negative Symptom Scale (PANSS) [17], the Scale for the Assessment of Negative Symptoms (SANS) [18] and the Brief Psychiatric Rating Scale (BPRS) [19]. Average scores were 16 ± 7.6 (range 7–30) for the PANSS-P, 19.2 ± 6.7 (range 8–27) for PANSS-N, 35.5 ± 8.4 (range 27–52) for PANSS-G, 52.1 ± 23.1 (range 24–89) for SANS, and 44.9 ± 10.9 (range 33–62) for BPRS. Five of the patients were admitted for inpatient treatment for the first time, while the remaining had been previously hospitalized between one and three times. Nine subjects of the patient group were receiving stable doses of antipsychotic medication, one patient was unmedicated (chlorpromazine equivalents: 493.9 ± 430.6 mg). Three patients received only typical antipsychotics, one patient received only atypical antipsychotics and five patients received both typical and atypical antipsychotics. All subjects were right-handed as assessed by the Edinburgh Handedness Inventory [20].

### Stimuli and task

Subjects saw a random sequence of a total of 960 pictures of objects selected from four super-ordinate categories (forest animals, flowers, clothes, and furniture). Each super-ordinate category was represented by four different base-level concepts (*animals*: bear, wolf, deer, fox; *flowers*: rose, sunflower, orchid, tulip; *clothes*: jacket, pants, shoe, shirt; *furniture*: table, chair, sofa, closet), and each base-level concept was represented by 60 pictures of different exemplars. Stimuli were presented in a sequence, randomized across and within blocks. Subjects were asked to decide for each stimulus whether it was an artificial or a natural object, decision being indicated by pressing one of two alternative response buttons on a response pad placed on the subject's lap. Responses were made with the index and middle fingers of the right hand, with conditions counterbalanced across fingers. The series of 960 stimuli was divided into 3 blocks of 320 pictures each, preceded by 16 additional practice trials that served to familiarize the subject with the task and the stimuli. Stimulus exposure time for each picture was 750 ms and the stimulus onset asynchrony was 1500 ms. A fixation cross was presented during the inter-stimulus intervals.

### Data acquisition and analysis

Neuromagnetic activity was recorded using a whole-head neuromagnetometer (MAGNES 2500 WH, 4D Neuroimaging, San Diego CA) installed within a magnetically shielded room (Vaccumschmelze, Hanau). The measuring surface of the sensor is helmet-shaped and covered the entire cranium, with the 148 sensors (magnetometer type) being arranged in a uniformly distributed array. Stimuli were presented via a mirror system.

Data were sampled at 678 Hz using a bandwidth of 0.1 to 200 Hz. After artifact-correction by noise reduction, correction of ocular and cardiac artifacts, and offline digital filtering at 0.1–30 Hz, the data were segmented in epochs of 1000 ms, starting 100 ms before stimulus onset, and baseline corrected. For every subject, the event-related response was averaged separately for each of the 16 base-level concepts and there was no significant difference between the two groups in the number of trials that entered the further analyses (t(18) = 1.01; n.s.). The grand mean across all concepts was subtracted from the grand mean of each single concept to remove activity not related specifically to it. Activity in the source space was determined by the minimum norm estimate including 197 dipoles located on a sphere [21]. This distributed source model provides the best estimate of the sources underlying the extracranially recorded magnetic field when minimal a priori information about these sources is available. Spatial activation in the source space was compared separately for 17 cortical areas. Based on theoretical considerations as well as empirically observed peaks of neuromagnetic activity, the analysis was focused on two time epochs, 170–210 ms, and 210–450 ms following stimulus onset (the first interval representing initial stages of visual processing [22], the second being associated with semantic activity).

**Figure 1:**
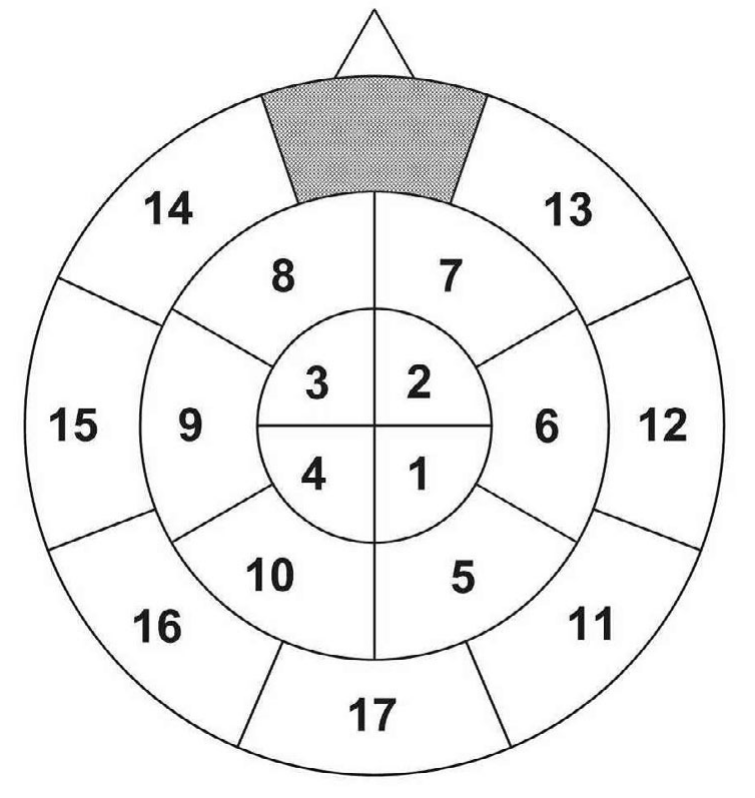
Schematic representation of the distribution of the 17 areas clustered for analysis.

Associations were evaluated as similarities among the patterns of neuromagnetic activity elicited by each base-level concept. Pearson correlation coefficients for all possible pairs of the 16 base-level concepts were determined for the 17 areas (see Figure 1) across 8 dipole locations in each area. Coefficients were Fischer-Z-transformed and averaged to yield two data points per subject for each area, one representing the mean level of correlation for pairs of base-level concepts within the same super-ordinate category, and the other representing the mean level of correlation for pairs of base-level concepts across different categories. The difference between z-scores (between minus within categories) was calculated as a *contrast score *for each of the 17 cortical areas for the two time intervals specified above. These contrast scores capture the distinction between different semantic categories and the coherence of brain activity within each category. The distribution of the contrast scores provides information about the brain regions involved in this distinction.

Furthermore, an unsupervised clustering analysis was realized using a deterministic algorithm that, in sequential passes, clusters the two Euclidian closest high-dimensional vectors, so that at each pass the number of clusters is decreased by one [23]. After a cluster is formed, it is represented by the mean of the composing vectors. In this context, the clustering was considered successful if at one of the passes all of the 16 base-level concepts were clustered according to the 4 super-ordinate categories. The success of unsupervised clustering was determined from the number of unsuccessful clusterings, that is, deviancies from original base-level-concept vectors.

Group differences were evaluated by analyses of variance with the between factor GROUP and the within factors HEMISPHERE (left/right), GRADIENT (central/posterior) and ROW (dorsal/ventral) for the contrast scores for 8 selected areas covering temporal regions in both hemisphere, and the unsupervised clustering scores.

Effects of task difficulty and compliance were controlled by an analysis of variance including performance measures (reaction time and errors in man-made vs. nature-made decision). Effects of the psychopathological state on brain activation indices were evaluated by correlation analyses.

## Results

### Performance

Independent of category, schizophrenic patients responded slower (median RT: 605.4 ± 41.9 ms) than control subjects (557.0 ± 60.7 ms; main effect GROUP (F(1,18) = 4.31; p = 0.05), but did not make more errors than controls (93.1% vs. 93.9% correct responses, F < 1).

### Brain activation patterns

The pattern of spatiotemporal correlations, i.e., similarity of activity patterns for concepts within vs. across super-ordinate categories across time, differed between control and schizophrenic subjects. The contrast scores within vs. across categories were higher in controls than in patients (main effect GROUP, F(1,18) = 4.85, p < .05, for the time window 170–210 ms, F(1,18) = 5.22, p < .05 for 210–450 ms).

**Figure 2:**
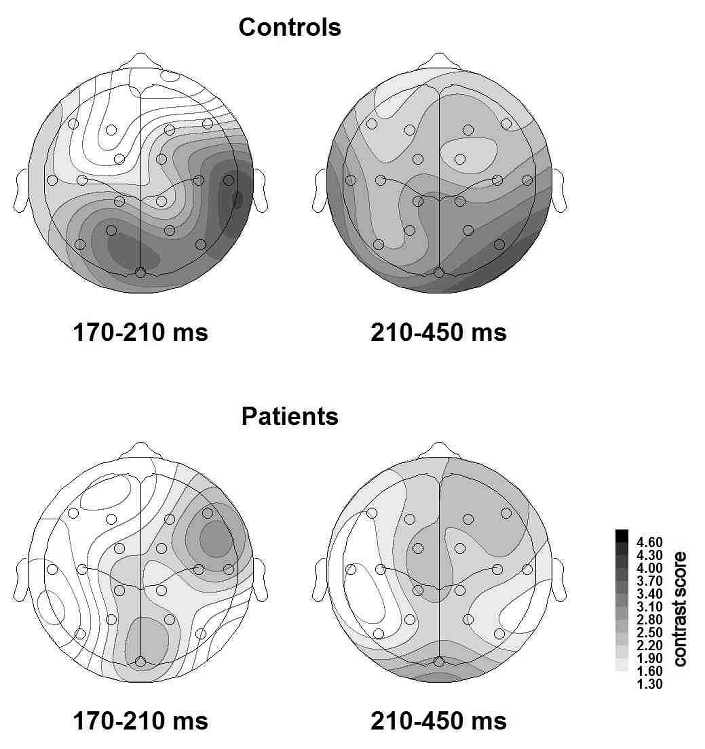
**Topographical distributions of the difference of the Z scores for correlations within and across categories for both time intervals and groups**. Left panel: 170–210 ms, right panel: 210–450 ms; top: controls, bottom: patients. Higher values indicate better discrimination between categories.

Figure 2 illustrates the topographical distributions of the contrast scores for both groups and time windows. In both groups, within-category coherence and across categories distinction 170–210 ms after stimulus onset were generally greater in right temporal areas (HEMISPHERE, F(1,18) = 5.94, p < .05; HEMISPHERE × anterior-posterior GRADIENT interaction, F(1,18) = 8.71, p < .01). Group differences were prominent in the bilateral temporal regions (areas 11, 12, 15, 16: main effect of GROUP F(1,18) = 6.94, p < .05; area-specific comparisons: area 11 (RH), (F(1,18) = 5.22, p < .05; area 16 (LH), F(1,18) = 5.14, p < .05). In the later time window (210–450 ms) schizophrenic patients displayed lower contrasts than control subjects in those temporal areas that are associated with the visual ventral processing stream (main effect GROUP × dorsal-ventral ROW, interaction F(1,18) = 4.27, p < .05; GROUP F(1,18) = 6.29, p < .05; area-specific comparisons: area 11: F(1,18) = 5.86; area 15: F(1,18) = 4.49, p < .05).

In the patient group, the distinctness of the categorical representation during the 170–210 ms epoch varied with symptom severity. Positive symptoms (according to the PANSS-P) tended to be associated with weaker contrasts in area 8: r = -.62, p < .1 and area 17: r = -.60, p < .1, while negative symptom were associated with higher contrasts (area 7: r = .81, p < .01; area 1: r = .69, p < .05). There was a trend for elevated formal thought disorder (indicated by 'conceptual disorganization' in the PANSS) to be related to weaker contrasts (area 17; r = -.62, p < .1).

Moreover, correlation coefficients between the contrasts and the PANSS-P were negative in 15 of the 17 areas, but were positive with the PANSS-N. Thus, more pronounced positive symptoms varied with blurred contrasts, but more pronounced negative symptoms with more distinct contrasts.

### Unsupervised clustering

**Figure 3:**
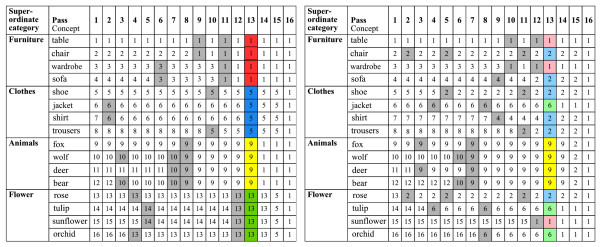
**Dynamic process of unsupervised hierarchical clustering of the data averaged between 210–450 ms over the left hemisphere**. For illustration, each base-level concept was assigned a sequential number from 1 to 16. Each column matrix represents the ad-hoc clustering at each pass. This process starts with 16 different vectors and ends with one collapsed cluster. Shaded cells highlight the online clustering of base-level concepts within one super-ordinate category. Note that after 12 passes, the clustering accurately reflects the superordinate categories for the controls (left), but not for the patient group (right).

Figure 3 illustrates the results of unsupervised clustering based on the averaged data for the two groups: for controls, the base-level concepts were clustered in their four apriori super-ordinate categories after 12 passes, while for the patient group 'mis-allocations' were frequent. In contrast to the control group, none of the supra-ordinate categories was correctly clustered in the schizophrenic group. Clustering based on individual data revealed a significantly higher number of 'mis-allocations' after 12 passes in patients (10.1 of 16 base concepts) than in controls (6.3 of 16 base concepts, GROUP, F(1,18)= 12.38, p < .01). Moreover, the algorithm determined more 'mis-allocations' across categories (e.g. clustering vectors from category 1 into cluster 4) for schizophrenics (on average 2.3) than for control subjects (on average 1.5, t(18) = 2.87, p < .05). None of the patients exhibited two correct clusters representing two of the original categories after 12 passes compared to an average of 2.3 in controls (t(18) = 4.48, p < .01).

## Discussion

The present results indicate different cortical representations of object categories and their brain organization in schizophrenic patients compared to normal control subjects. In control subjects, the macroscopically measured brain MEG responses in the temporal cortex reflect considerable associative strengths within categories with a clear distinction across supra-ordinate categories. In schizophrenia patients this measure of associative strength is significantly weaker, although some categorical grouping is represented in the same brain region as in controls. Consistent with the role of the ventral visual stream as the major anatomical substrate of object representation [24], the reduced distinctiveness in the categorization of objects was found in areas corresponding to the ventral processing stream, where the main intergroup differences were also found. This "loosening" of associative relations is supported by unsupervised hierarchical clustering. This analysis confirmed an ill-defined organization of the 16 base-level concepts in 4 a-priori defined super-ordinate concepts in schizophrenic patients compared to control subjects. The failure to achieve sufficient clustering strengthens the assumption of weak borders between representations in psychotic associative networks.

Although limited by sample size, there was a suggestion that the severity of positive symptoms was related to less orderly categorization as indicated by weaker contrast scores. Interestingly, the disturbed object processing in schizophrenia is already seen within the first 200 ms and associations with clinical symptoms were only found during the earlier time window. This time course supports previous findings that suggested deficits in semantic processing in schizophrenia during early automatic activation [25,26]. Alternative explanations of these results should, however, be considered:

For example, these results might reflect a higher level of "noise" in the schizophrenic data compared to the control group, a smaller number of trials or more artifacts in the schizophrenia sample. Note, however, that there was no difference between groups in the number of trials included in the analysis and artifacts were carefully corrected. In addition, the average z-transformed correlation in the schizophrenia sample was not different from that of the control group. Rather the similarity of brain responses to the different items did not consistently follow the objective categories in the schizophrenia sample. A second alternative account could be that the small number of subjects might have reduced the power of the results. Nevertheless, statistical differences between the groups have been obtained, even though schizophrenic patients varied in their symptom profiles. Hence a more plausible explanation is that the abnormality in semantic categories and the related "loosening of associations" is part of the set of common base dysfunctions, which "surfaces" in various symptoms. Psychosis can be viewed as a manifestation of a maladaptive brain organisation that arises from unfavourable interaction of genome and environment. The present results provide further support to assume an abnormal cerebral network architecture with altered neural connectivity and communication at the core of this dysfunction. It seems plausible that these changes lead to neuropsychological, cognitive and behavioural malfunctioning that give rise to the psychiatric symptoms on the subjective and behavioural level. Given further development in brain imaging, there is, however, no longer a need to rely exclusively on "surface" measures for classification of disease and diagnosis. It has become possible to directly map dysfunctional neural networks in the brain by recording, for example, abnormal magnetic brain activity [27]. Enhanced slow wave activity is found in the majority of psychotic patients [28] and has been viewed as a dysfunction in association cortex. The present findings indicate that with it, the functional organisation in these structures has become disordered. Work from Bao et al. [8] would predict that imbalance in the dopaminergic system may have such consequences. If true, proper neuroleptic and anticholinergic medication may be essential to provide the basis for ordered categorical representations, although it might not be sufficient to build up or restore such representations. This would require corresponding systematic training of the base-level concepts.

## Conclusion

The present study illustrates how mapping of higher-order categorical representation and of dysfunctional neural networks may help us to understand psychotic disorders from a neuroscience perspective. Spatio-temporal correlations and hierarchical clustering revealed a less organized representation of semantic categories in clusters of magnetic brain responses in schizophrenic patients compared to healthy adults. This basic deficit of psychotic neural network architecture may be a correlate of loosening of associations, promoting positive symptoms.

## Competing interests

The authors are employees of the University of Konstanz (BR, TE), the Hebrew University Jerusalem (SB, YS), or research fellow at the NIMH Center for the Studies of Attention and Emotion at the University of Florida, Gainesville (AL). Publication of the paper does not affect interests, financial or otherwise, of any organization, neither employment nor consultancy or shared ownership, of any of the authors.

## Authors' contributions

The study was designed by AL, BR, TE and SB. Data were acquired by AL and analyzed by AL and YS. AL, BR, TE and SB drafted the manuscript, all authors revised the manuscript and approved the final version.

## Pre-publication history

The pre-publication history for this paper can be accessed here:


